# Antibacterial effects of *Apis mellifera* and *stingless* bees honeys on susceptible and resistant strains of *Escherichia coli*, *Staphylococcus aureus* and *Klebsiella pneumoniae* in Gondar, Northwest Ethiopia

**DOI:** 10.1186/1472-6882-13-269

**Published:** 2013-10-19

**Authors:** Yalemwork Ewnetu, Wossenseged Lemma, Nega Birhane

**Affiliations:** 1 Department of Biotechnology, Natural and Computational Sciences Faculty, University of Gondar, Gondar, Ethiopia; 2 Department of Parasitology, School of Biomedical and Laboratory Sciences, College of Medicine and Health Sciences, University of Gondar, Gondar, Ethiopia

**Keywords:** Anticbacterial effects, Ethiopian honeys, *Escherichia coli* (ATCC 25922), *Escherichia coli* (R), *Klebsiella pneumoniae* (R), *Staphylococcus aureus* (ATCC 25923), *Staphylococcus aureus* (MRSA)

## Abstract

**Background:**

Honey is a natural substance produced by honeybees and has nutritional and therapeutic uses. In Ethiopia, honeys are used traditionally to treat wounds, respiratory infections and diarrhoea. Recent increase of drug resistant bacteria against the existing antibiotics forced investigators to search for alternative natural remedies and evaluate their potential use on scientific bases. Thus, the aim of this study was to evaluate the antibacterial effects of different types of honeys in Ethiopia which are used traditionally to treat different types of respiratory and gastrointestinal infections.

**Methods:**

Mueller Hinton agar (70191) diffusion and nutrient broth culture medium assays were performed to determine susceptibility of *Staphylococcus aureus* (ATCC 25923), *Escherichia coli* (ATCC 25922) and resistant clinical isolates (Methicillin resistant *Staphylococcus aureus*(MRSA), *Escherichia coli*(R) and *Klebsiella pneumoniae* (R), using honeys of *Apis mellifera* and stingless bees in northern and north western Ethiopia.

**Results:**

Honey of the stingless bees produced the highest mean inhibition (22.27 ± 3.79 mm) compared to white honey (21.0 ± 2.7 mm) and yellow honey (18.0 ± 2.3 mm) at 50% (v/v) concentration on all the standard and resistant strains. Stingless bees honey was found to have Minimum Inhibitory Concentration (MIC) of 6.25% (6.25 mg/ml) for 80% of the test organisms compared to 40% for white and yellow *Apis mellifera* honeys. All the honeys were found to have minimum bactericidal concentration (MBC) of 12.5% (12.5 mg/ml) against all the test organisms. *Staphylococcus aureus* (ATCC 25923) was susceptible to amoxicillin, methicillin, kanamycine, tetracycline, and vancomycine standard antibiotic discs used for susceptibility tests. Similarly, *Escherichia coli* (ATCC 25922) was found susceptible for kanamycine, tetracycline and vancomycine. *Escherichia coli* (ATCC 25922) has not been tested for amoxicillin ampicillin and methicillin. The susceptibility tests performed against *Staphylococcus aureus* (MRSA), *Escherichia coli* (R) and *Klebsiella pneumoniae* (R) using three of methicillin, erythromycin, ampicillin, Penicillin and amoxicillin discs were resistant. But, these drug resistant strains were susceptible to antibacterial agents found in the honeys and inhibited from 16 mm to 20.33 mm.

**Conclusions:**

Honeys in Ethiopia can be used as therapeutic agents for drug resistant bacteria after pharmaceutical standardization and clinical trials.

## Background

Honey is a natural substance produced and stored in the honeycombs by honeybees with carbohydrates constituting about 95 to 97% of the dry weight of honey [1,2]. Fructose and glucose are the most predominant sugars present and responsible for most of the physical and nutritional characteristics of honey [3,4]. The volatile compound found in honey includes alcohols, ketones, aldehydes, acids, esters and terpenes [5]. Phenolic acids (benzoic and cinnamic acids) and flavonoids (flavanones, flavanols) contribute significantly to the therapeutic capacity of honey which varies greatly depending on the floral source [6]. Different studies have shown honey to have antimicrobial effect [7-11], anti-inflammatory effects [12], anti-oxidant effects [13] and boosting of the immune system [14]. Common Apinae honey bees honey (*Apis mellifera* honey) and stingless honeybees honey are the two types of honeys found in the world [15]. The common honey bees (sub family Apinae) and stingless bees (sub family Meliponinae) are grouped in the family Apidae [16]. In Ethiopia, Apinae honeybees (*Apis mellifera*) are mostly domestic unlike the wild stingless honeybees which keep their honey in storage pots build of resinous cerumen in the ground (“Tazma” honey) or in the tree trunk (“Tinign” honey). Tazma and Tinign honeys are the same as stingless bees could nest in the ground or tree trunks depending their preferences. The different species of stingless bees and their behaviour were not studied in Ethiopia, although *Apis mellifera* and stingless bees honeys have been tested for antimicrobial activities against different bacteria [7,9-11] in addition to traditional use of these honeys to treat wounds, respiratory infections and diarrhoea. At present, Medihoney TM (a blend of manuka and jelly bush honey) has been one of the first medically certified honeys licensed as medical product for professional wound care in Europe, America and Australia [17]. Honey has shown considerable antibacterial activity against a wide range of wound pathogens [18] as well as against biofilms created by bacteria on wounds [19].

The antimicrobial (antibacterial, antiviral, antifungal and antiparasitic) activities of honeys were reported due to high osmolarity, acidity, hydrogen peroxide and phytochemicals [17,18,20-25]. The major antibacterial effect, however, was reported due to hydrogen peroxide [15,26-29]. Hydrogen peroxide is produced by the oxidation of glucose by the enzyme glucose-oxidase (Glucose + O_2_**→** gluconic acid + H_2_O_2_) [30]. The peroxide activity in honey can be destroyed easily by heat or the presence of catalase in the body tissues and serum [19]. *In vivo* use of honey for human as therapeutic agent, therefore, requires the evaluation of non-peroxide phytochemical components of honey. The non-peroxide phytochemical components’ of Manuka Apinae honey (after removing hydrogen peroxide by treating with enzyme catalase) from New Zealand has been found to have substantial levels of antibacterial activity [31]. Such manuka honey was tested against seven species of bacteria and was found to have MIC (minimum inhibitory concentration) that range from 1.8% to 10.8% (v/v) [32]. This result, therefore, indicated that the major antimicrobial effect of honey may not be due to hydrogen peroxide. Propolis (resinous protective barrier) in stingless bees honey has been reported to have therapeutic effects against inflammation, heart disease, diabetes mellitus, microbe’s hepatotoxity, and cancer [27,29]. In agar diffusion, 1% ethanol extracts of propolis were found inhibitory for growth *S. aureus*, *E. coli*, and *P. aeruginosa*[33]. When *Apis mellifera* honey (eucalyptus) and stingless bees honey were compared, the former had higher phenolic and flavonoid contents than the stingless bees’ honey, which in turn had the higher antioxidant activity [34]. The aim of this study was to evaluate the antibacterial effects of Ethiopian honeys against antibiotic susceptible and resistant strains of bacteria so that they would have been recommended as therapeutic agents after pharmaceutical standardization and clinical trials.

## Methods

### Study area and period

Gondar town is located in Amhara region, North West Ethiopia, at about 723 kms from Addis Ababa. It is located, at an altitude of around 2, 225 m (above sea level), 12°35′60.000”N of latitude and 37°28’0.120”E of longitude. White *Apis mellifera* honey from Tigray (Northern Ethiopia), yellow *Apis mellifera* honey from Gondar (northwest Ethiopia) and Stingless honey from Gojam (northwest Ethiopia) were purchased in September and October, 2012 G.C and their antibacterial effects were analyzed in biotechnology laboratory in University of Gondar from September 20, 2012 to January 1, 2013 G.C. Only the stingless bees honey collected from the ground (Tazma honey) was used for susceptibility test. Attempts were not made to obtain the stingless bees honey from tree trunks. Field observation has showed absence of difference between the two types of honeys despite the difference in nesting sites.

### Test organisms

*Staphylococcus aureus* (ATCC 25923), *Escherichia coli* (ATCC 25922), Staphylococcus *aureus* (MRSA), *Escherichia coli* (R) and *Klebsiella pneumoniae* (R) used in this study were obtained from Gondar University teaching hospital laboratory.

### Preparation of honey solutions

Hundred percent pure honeys (100% v/v) was obtained after the stingless bees and *Apis mellifera* honeys were filtered using sterile gauze. To get 50% honey solutions (v/v), 1 gm honey was diluted in 1 ml distilled water. Further serial dilutions were done to obtain 25% and 12.5% honey solutions (v/v).

### Preparation of the Mueller Hinton agar (MHA)

Mueller Hinton agar (70191) medium was prepared by dissolving 38 g of Mueller Hinton agar in 1000 ml distilled water and boiled until complete dissolutions. The solution was sterilized in an autoclave (121°C, 1 bar) for 15 min. The suspension was poured (20 ml) into sterile petri-dishes in the hood to solidify at room temperature.

### Preparation of the nutrient broth (7146)

After dissolving 8 g nutrient broth powder in one liter of purified water, the mixture was mixed thoroughly to form a clear medium which will be incubated at 35°C for 18 – 24 hours after the bacterial specimens were inoculated. Turbidity indicates good growth. Nutrient broth culture medium can live longer under refrigeration.

### Preparation of 0.5 McFarland standards

In this study, 0.5 ml of 0.048 M BaCl_2_ (1.175%W/V BaCl_2_.2H_2_O) was added to 99.5 mL of 0.18 M H_2_SO_4_ (1% V/V) with constant stirring to make 0.5 McFarland Standards [35]. The standard was distributed into a screw capped test tube of the same size and volume as those used to prepare the test inoculums. Hundred micro-liter (100 μl) bacteria sample from nutrient broth culture media (lot Himedia laboratory, pvt, ltd, India) was added into 5 ml saline and the concentration was adjusted to 1–2 ×10^8^ colony forming unit per mill liter (Cfu/ml) by comparing with McFarland 0.5 standardized [35].

### Preparation of inoculations and assays of antibacterial activities

The inoculation of the bacteria was done by streaking the surface of the plates with sterile swab in a zigzag manner until the entire surface was covered. With a previously sterilized cork borer (4 mm) size, wells of equal distance were bored to drop 100 μl of different antimicrobial agents (honeys). Hundred micro liters (100 μl) of 50%, 25% v/v and 12.5% of the honey solutions were inoculated in to wells of *Escherichia coli* (ATCC 25922), *Staphylococcus aureus* (ATCC 25923), Staphylococcus *aureus* (MRSA), *Escherichia coli* (R) and *Klebsiella pneumoniae* (R). The culture plates were incubated at 37°C for 24 h. Inhibition zones were indicated by clear area around the wells which were measured in millimeters by caliper in order to evaluate the degree of susceptibility of the test organisms for 50%, 25% and 12.5 solutions (v/v). This susceptibility test against the different honeys solutions were repeated three times to use an average results.

### Minimum inhibitory concentration (MIC) and minimum bactericidal concentration (MBC)

Hundred micro-liter (100 μl) bacteria samples from nutrient broth culture medium were added into 5 ml saline and the concentration was adjusted to 1–2 ×10^8^ Cfu/ml by comparing with McFarland 0.5 standard with constant stirring before culturing in new broth medium to determine the lowest concentration of antimicrobial agent capable of preventing growth (Minimum inhibitory concentrations (MIC)).

The inoculations of *Escherichia coli* (ATCC 25922), *Staphylococcus aureus* (ATCC 25923), Staphylococcus *aureus* (MRSA), *Escherichia coli* (R) and *Klebsiella pneumoniae* (R) were done in different nutrient broth medium containing different concentrations (50%, 25%, 12.5% and 6.25% solutions v/v) of honey solutions. The tubes were incubated for 20–24 hours at 37°C to observe turbidity (growth) which indicated the MIC of the different honeys on the test organisms.

The minimum bactericidal concentrations (MBCs) were determined by sub culturing the contents of nutrient broth used for MIC tests on Mueller Hinton agar media using sterile wire loop and making a strike on the media to see bacteria growth after incubating at 37°C for 24 hours. Absence of growth indicated the minimum bactericidal concentrations (MBCs) of the honeys.

### Drug susceptibility

Drug susceptibility of *Staphylococcus aureus* (ATCC 25923), Staphylococcus *aureus* (MRSA), *Klebsiella pneumoniae* (R), *Escherichia coli* (ATCC 25922) and *Escherichia coli* (R) cultures were determined using at least three of the following antibiotics discs: Tetracycline, vancomycin, amoxicillin, methicillin, Erythromycin and penicillin. The result was interpreted as resistant, intermediate or susceptible by comparing the results with what has already been reported by Clinical and Laboratory Standards Institute (CLSI) [36].

### Statistical analysis

The antibacterial effects (inhibitions) of honeys (mean ± SD) were compared using descriptive statistics. All statistical analysis has been performed by using statistical package of social science (SPSS) version 20. Comparison of honey extracts, for their mean inhibitions, were analyzed using one way analysis of variance (ANOVA). Mean inhibitions of the different honey solutions were considered significantly different for P value less than 0.05.

## Results

**Figure 1:**
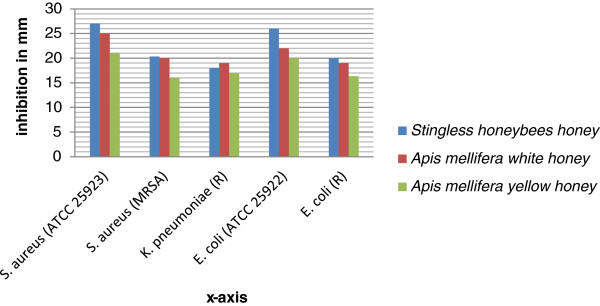
**Bacteria inhibitions of 50% solution (v/v) of stingless bees honey, Apis mellifera white and yellow honeys on *Staphylococcus aureus *** **(ATCC 25923), *Staphylococcus aureus *** **(MRSA), *Klebsiella pneumoniae *** **(R), *Escherichia coli *** **(ATCC 25922) and *Escherichia coli *** **(R).**

The overall mean inhibitions of the 50% honey concentrations (v/v) were higher compared with 25% and 12.5% concentrations on *Staphylococcus aureus* (ATCC 25923), *Staphylococcus aureus* (MRSA), *Escherichea coli* (ATCC 25922), *Escherichia coli* (R) and *Klebsiella pneumoniae* (R) (Table 1). The highest inhibition (27 mm) was produced by stingless bees honey on both susceptible *E. coli* (ATCC25922) and *S. aureus* (ATCC25923). Of all the three types of honeys, the stingless bees honey produced the leading inhibitions on almost all the standard and resistant test organisms. *Apis mellifera* yellow honey produced the least effect (Figure 1). A one-way between-groups analysis of variance using Post-hoc comparisons using the Tukey tests have shown statistically significant mean differences at the p < 0.05 level for inhibitions of stingless honey bees honey (Mean *±* SD = 22.27 ± 3.78, df = 14, F *=* 44.59*,* P <0.05), white honey ( 21 ± 2.69, df = 14, F *=* 8.13*,* P < 0.05) and yellow honey ( 18 ± 2.31, df = 14, F *=*12.29, P < 0.05 ) on *Staphylococcus aureus* (ATCC 25923), *Staphylococcus aureus* (MRSA), *Escherichea coli* (ATCC 25922), *Escherichea coli* (R) and *Klebsiella peumoniae* (R) at 50% concentration (v/v) (Table 2). Similarly, statistically significant mean differences were observed for 25% and 12.5% concentrations (v/v). Stingless bees Tazma honey have 6.25% (6.25 mg/ml) MIC value for 80% of the test organisms compared to 40% for *Apis mellifera* white and yellow honeys (Table 3). All the honeys were found to have 12.5% (12.5 mg/ml) Minimum Bactericidal Concentration (MBC).

**Table 1:** **Mean inhibition and standard deviation of stingless bees and *Apis mellifera *** **honeys on *Staphylococcus aureus *** **(ATCC 25923), *Staphylococcus aureus *** **(MRSA), *Klebsiella pneumoniae *** **(R), *Escherichia coli *** **(ATCC 25922) and *Escherichia coli *** **(R) at 50% (v/v) concentration**

**Conc. (v/v)**	**Type of honey**	**N**	**Minimum**	**Maximum**	**Mean**	**Std. deviation**
50%	Stingless honeybees honey	15	16.00	27.00	22.26	3.79
	*Apis mellifera* white honey	15	17.00	25.00	21.00	2.70
	*Apis mellifera* yellow honey	15	14.00	21.00	18.07	2.31
25%	Stingless honeybees honey	15	15.00	21.00	18.73	1.83
	*Apis mellifera* white honey	15	9.00	18.00	13.67	3.29
	*Apis mellifera* yellow honey	15	7.00	16.00	10.40	3.58
12.5%	Stingless honeybees honey	15	9.00	19.00	14.40	2.82
	*Apis mellifera* white honey	15	8.00	17.00	12.40	3.33
	*Apis mellifera* yellow honey	15	6.00	15.00	9.67	3.06

**Table 2:** **Results of one way ANOVA from the inhibitions of the three types of honeys on *Staphylococcus aureus *** **(ATCC 25923), *Staphylococcus aureus *** **(MRSA), *Klebsiella pneumoniae *** **(R), *Escherichia coli *** **(ATCC 25922) and *Escherichia coli *** **(R) showing mean difference for 50% solutions (v/v)**

**Type of honey**		**Sum of squares**	**Degrees of freedom (df)**	**Mean square**	**F**	**Sig.**
Stingless honey bees honey	Between Groups	190.27	4	47.57	44.59	.000
	Within Groups	10.67	10	1.07		
	Total	200.93	14			
*Apis mellifera* white honey	Between Groups	78.00	4	19.50	8.13	.003
	Within Groups	24.00	10	2.40		
	Total	102.00	14			
*Apis mellifera* yellow honey	Between Groups	62.27	4	15.57	12.29	.001
	Within Groups	12.67	10	1.27		
	Total	74.93	14			

**Table 3:** **Minimum inhibitory concentration (MIC) values of stingless honey, Apis mellifera white honey and Apis mellifera yellow honey against drug susceptible (*Staphylococcus aureus *** **(ATCC 25923) and *Escherichia coli *** **(ATCC 25922)) and resistant clinical isolate (*Staphylococcus aureus *** **(MRSA), *Klebsiella pneumoniae *** **(R), and *Escherichia coli *** **(R)) at 50% (v/v) concentration**

**MIC(%) of the different bactericidal agents against the test organisms**			
**Test organisms**	**Stingless honey bees honey**	**Apis mellifera white honey**	**Apis mellifera yellow honey**
E.coli(S)	6.25	6.25	6.25
S. aureu(S)	6.25	6.25	6.25
E.coli(R)	6.25	12.25	12.25
K. pn (R)	6.25	12.25	12.25
S. au(R)	12.25	12.25	12.25
% at 6.25	80%	40%	40%
% at 12.5	20%	60%	60%

Tetracycline, Amoxicillin and Kanamycin standard antibiotic discs produced 22 mm, 25 mm and 20 mm inhibition zones on susceptible *Staphylococcus aureus* (ATCC 25923) respectively. Susceptible *Escherichia coli* (ATCC 25922) also inhibited by tetracycline (21 mm), kanamycin (20 mm) and erythromycin (10 mm) standard antibiotic discs. Tetracycline and methicillin antibiotic discs did not produce inhibition on *Staphylococcus aureus* (MRSA) while amoxicillin produced very low (10 mm) inhibition. Methicillin disc produced no inhibition on resistant *E. coli.* Similarly, Ampicillin, erythromycin and methicillin did not produce inhibitions on resistant *Klebsiella pneumoniae* (R) strain (Table 4).

**Table 4:** **Antibacterial effect (susceptible, intermediate and resistant) of Tetracycline (30 μg), Kanamycine (30 μg), Erythromycin, Methicillin (5 μg), Ampicillin (10 μg), Amoxicilline (25 μg), Penicillin (10 μg) and Vancomycine (30 μg) antibiotic discs on drug susceptible (*Escherichia coli *** **(ATCC 25922)** & ***Staphylococcus aureus *** **(ATCC 25923) and resistant clinical isolate (*Staphylococcus aureus *** **(MRSA), *Escherichia coli *** **(R) and *Klebsiella pneumoniae *** **(R)**

**Antimicrobial drugs**	***S. aureus *** **(ATCC 25923)**	***S. aureus *** **(MRSA)**	***K. pneumoniae *** **(R)**	***E. coli *** **(ATCC 25922)**	***E. coli*** **(R)**
Amoxicillin (25 μg)	S	R	_	_	R
Ampicillin (10 μg)	_	_	R	_	_
Methicillin (5 μg)	I	R	R	_	R
Kanamycin (30 μg)	S	_	_	S	_
Penicillin (10 μg)	R	R	_	R	_
Tetracycline (30 μg)	S	_	_	S	_
Erythromycine	_	_	R	R	R
vancomycin (30 μg)	S	_	_	S	S

Keys: - S = susceptible; I = intermediate; R = resistant.

## Discussion

The inhibitions of *Apis mellifera* white Tigray honey (25 mm for *Staphylococcus aureus* (ATCC 25923) and 22 mm for *Escherichia coli* (ATCC 25922)) and stingless bees Gojam Tazma honey (27 mm for *Staphylococcus aureus* (ATCC 25923); 26 mm for *Escherichia coli* (ATCC 25922)) at concentration of 50% solution (w/v) were as effective as inhibitions of standard tetracycline discs already reported (83). In this study, the inhibitions produced by singless Gojam honey and *Apis mellifera* white Tigray honey on *Staphylococcus aureus* (ATCC 25923) and *Escherichia coli* (ATCC 25922) at 50% concentration (v/v) (Figure 1) was greater than inhibitions produced by amoxicillin (25 μg), Methicillin (5 μg), Kanamycin (30 μg), Penicillin (10 μg) and Tetracycline (30 μg) discs (Table 4). Unlike the honeys, Methicillin, erythromycin, ampicillin and amoxicillin discs did not produce significant antibacterial effects on the resistant strains of bacteria (Table 4). These results have indicated the potential of honeys as therapeutic agents to treat both susceptible and drug resistant bacteria. The inhibitions of the 25% concentration of stingless honeybees honey (Tazma) solutions (v/v) in this study were similar to previous study [10]. Antibacterial effects of honey solutions decreased up on serial dilution from 50% to 25% which has also been reported elsewhere [19].

Of the three types of honeys analyzed, stingless bees tazma honeys were found to have the highest antimicrobial activities against susceptible bacteria compared to *Apis mellifera* white Tigray honey (Table 1). The presence of bactericidal resins (propolis) might have given extra antimicrobial effect of stingless bees Gojam tazma honey as already reported [37]. The difference between *Apis mellifera* white and yellow honeys could be related to floral sources of honeys as already reported in another study [6]. *Apis mellifera* in Tigray often make their honey from white flower of Cactus plant (*Opuntia spp*) unlike Gondar region where *Apis mellifera* use different plants as source of honey. The difference in floral sources, in these two localities, might explain the difference in antibacterial effects of *Apis mellifera* Tigray white and Gondar yellow honeys.

MIC of White and yellow honeys for 40% of the test organisms was 6.25% (Table 3). But, Mulu *et al*., (2004) [8] reported MIC values to be 6.25% for 90% of the test organisms. This difference could be related to difference in contents of the honeys as the two researches were conducted in different parts of Ethiopia in addition to the difference in tested organisms. The MIC value for stingless bees Tazma honey was 6.25% (6.25 mg/ml) for 80% of test organisms which was slightly higher than previous report [10]. The difference could be related to difference in the floral contents used to prepare the honeys. The minimum bactericidal concentration for this study was 12.5% (12.5 mg/ml) for all the test organisms compared to 6.25% for 70% the test organisms in another study [8] which might have been related to the floral contents used to prepare the honeys.

The results of antibiotic tests in this study on the susceptible standard *Escherichia coli* (ATCC 25922) and *Staphylococcus aureus* (ATCC 25923) were within the acceptable range (Table 1). But, the resistant clinical isolates (*Staphylococcus aureus* (MRSA), *Escherichia coli* (R) and *Klebsiella pneumoniae* (R)) were not effectively inhibited by methicillin, erythromycin, ampicillin and amoxicillin discs indicating the wide spread of drug resistant strains in the Gondar teaching referral hospital. Similar wide spread of β-lactamase producing drug resistant bacteria strains were reported to exist in the Hospitals (6–8). The results of this study has shown the potential of *Apis mellifera* white Tigray honey and stingless bees Gojam Tazma honey to be used to treat β-lactamase producing drug resistant bacteria strains in Ethiopia. Further clinical trial and pharmaceutical standardization of honeys are recommended before any use of the honeys as therapeutic agents.

## Conclusion

In conclusion, all the three types of honeys were not the same in their antibacterial effects. The leading bacterial inhibitions were produced by stingless bees honey and followed by *Apis Mellifera* white honey. The fact that inhibitions of these honeys were superior, over the most common antibiotics used to treat bacteria, makes them a novel source of chemotherapeutic agents to treat drug resistant bacteria in future.

## Competing interests

The authors declare that they have no competing interests.

## Authors’ contributions

YE, WL and NB participated in selecting the study area and preparing the proposal. YE carried out the experiment as part of MSc thesis and NB supervised the overall activities and reviewed the documents. WL involved in statistical analysis and preparation of this manuscript. All authors have read and approved the final manuscript.

## Pre-publication history

The pre-publication history for this paper can be accessed here:

http://www.biomedcentral.com/1472-6882/13/269/prepub
